# Enhancing Medical Students’ Disease Detection Skills: Leveraging Artificial Intelligence Software for Diagnosing Developmental Dysplasia Using Ultrasound Imaging

**DOI:** 10.3390/diagnostics16193093

**Published:** 2026-09-24

**Authors:** Yu-Ran Heo, Jun-Chae Lee, Yeon-Kyoung Ko, Seung-Bo Lee, Jae-Ho Lee, Si-Wook Lee

**Affiliations:** 1 Division in Anatomy and Developmental Biology, Department of Oral Biology, Human Identification Research Institute, BK21 FOUR Project, Yonsei University College of Dentistry, Seoul 03722, Republic of Korea; 2 Medical Course, Keimyung University School of Medicine, Daegu 42601, Republic of Korea; chai1703@naver.com; 3 Department of Medical Informatics, Keimyung University School of Medicine, Daegu 42601, Republic of Korea; 4 Department of Anatomy, Keimyung University School of Medicine, Daegu 42601, Republic of Korea; 5 Department of Orthopedics, Keimyung University Dongsan Medical Center, Daegu 42601, Republic of Korea; 6 MaiT Co., Ltd., Daegu 41931, Republic of Korea

**Keywords:** artificial intelligence, developmental dysplasia, medical education, ultrasonography

## Abstract

**Background:** Ultrasound is the gold standard for the screening of developmental dysplasia of the hip (DDH). However, there are several problems in overcoming the learning curve of technical skills required to detect DDH using ultrasound. Thus, several attempts have been made to assist in the detection of DDH using artificial intelligence (AI) software (SW). This study aimed to evaluate the efficacy of AI in DDH ultrasound screening to aid medical students in the diagnosis of DDH. **Method:** A Mask R-CNN-based detection model was developed to identify six key points and measure the alpha and beta angles, as well as containment, following Graf’s method. Fifty-three second-year medical students assessed 38 DDH screening ultrasound images from 28 patients twice: first through self-measurement after brief training and then using AI-assisted measurement, where key points were refined based on AI predictions. The gold standard for comparison was the measurement performed by an experienced orthopedic surgeon. **Results:** AI assistance significantly improved agreement with the expert reference for all three measurements. The proportion of students classified in the excellent agreement category increased from 26.4% to 81.1% for the alpha angle, from 67.9% to 86.8% for the beta angle, and from 56.6% to 84.9% for containment. The mean ICC increased from 0.593 to 0.855 for the alpha angle, from 0.760 to 0.892 for the beta angle, and from 0.722 to 0.882 for containment. Wilcoxon signed-rank tests demonstrated significant improvements in all three measurements (all *p* < 0.001). **Conclusions:** AI-assisted ultrasound measurement may improve agreement with expert measurements among novice users assessing DDH. This approach may have potential as a supportive tool for DDH screening and educational training by reducing the learning curve associated with ultrasound-based measurement.

## 1. Introduction

Developmental dysplasia of the hip (DDH) is one of the most common developmental disorders of the hip. It ranges from delayed physiological development of the hip to dislocation [1,2]. The true incidence of DDH has been difficult to estimate, and reported incidence ranges from 1:100 to 1–28:1000 newborns. Recently, large-scale ultrasound screening studies have indicated that ultrasound-detectable abnormalities may occur in 5–7% of all newborns [3]. Diverse factors such as breech presentation, oligohydramnios, and swaddling may be risk factors for DDH [1,4]. The earlier the detection, the better the prognosis of DDH. Ultrasound is usually used in newborns and infants because of the risk of radiation [2,4]. As ultrasound images are typically of low resolution and grayscale, it is difficult for unskilled operators to diagnose DDH using ultrasound image [5,6,7]. Thus, many studies using artificial intelligence (AI) software (SW) are being conducted for various diseases in which diagnosis is technique-dependent. AI technology can be trained to use pattern recognition to highlight suspicious features in images and flag them to aid clinicians in the diagnosis. It aims to reduce diagnostic errors made by clinicians [8,9]. Previous studies have indicated that an AI system with an accuracy of 87.9% can help ultrasound operators diagnose rotator cuff lesions and has a positive effect on the Cobb angle measurement and scoliosis diagnosis on chest radiographs [10,11]. Furthermore, AI has provided statistically significant assistance in the diagnosis of DDH using computed tomography images [12,13,14]. A few studies have shown that AI provides statistically significant assistance in the diagnosis of DDH using ultrasound [15,16]. In previous studies, Graf’s method was used for the diagnosis of DDH; if the alpha angle was more than 60°, it was defined as normal, and if it was less than 60°, it was defined as diseased. In addition, the beta angle and containment values were measured, and six key points were used to extract the corresponding values. Additionally, a scoring system was used to determine the quality of the ultrasound images used [7,15,17]. In this study medical students who participated used AI SW by MaiT^®^ for the diagnosis of DDH. This study aimed to evaluate the effectiveness of AI in DDH ultrasound screening to assist medical students in the detection of DDH.

## 2. Materials and Method

### 2.1. DDH Ultrasound Images

Data were collected between January 2016 and December 2021, after obtaining approval from the Institutional Review Board of Pusan University Hospital (PNU IRB no. 2301-013-123). This study was conducted in accordance with the Declaration of Helsinki. Among the 284 images obtained by radiologists from patients referred from primary care institutions or the Department of Pediatrics for DDH screening at Pusan National University, 9–10 points were extracted based on the modified 10-point scoring system through a purification process [15] (Figure 1). A total of 38 sheets were used. Of the 38 ultrasound images, 14 were from patients with DDH and 24 were from patients with normal hips.

### 2.2. DDH Screening Using AI SW

The AI model received ultrasound images as input and predicted six key points for DDH screening. The AI model was trained using 57,770 ultrasound images (Standard image training: validation: test = 20,752:2301:2557, nonstandard image training: validation: test = 26,056:2890:3214). The image was obtained from 1424 patients aged 0 to 18 months who visited Keimyung University Dongsan Hospital and 401 patients aged 0 to 8 months who visited Korea University Anam Hospital. The input images were automatically cropped, except for their regions of interest (ROI), to focus on the informative region and reduce unnecessary learning. To detect the six key points, a Mask R-CNN was used for human pose estimation. As the backbone of the Mask R-CNN, the feature pyramid network (FPN)-ResNet, a ResNet 101 model using the FPN method with a top-down architecture, was used. The anchor sizes were set to 32, 64, 128, 256, and 512, and the aspect ratio was set to 0.5, 1, and 2, which performed best as a result of a heuristic random search [18]. The learning rate started at 0.1 and was divided by 10 when the training loss was saturated. The weight decay and momentum were 0.0001 and 0.9, respectively. The Mask R-CNN-based DDH key point-detection model was trained for 100 epochs. The performance of the key point detection model was evaluated by 10-fold cross-validation of the training data and external validation data using the following evaluation metrics: object key point similarity, mean average precision, mean average recall, alpha error, and beta error for detecting the six key points. Alpha and beta errors are the differences between Graf’s alpha and beta angles measured using six real key points and six key points predicted by the key point detection model. A custom image review web application based on the Python Flask framework (public domain, http://mait.healthcare/ddh, accessed on 15 December 2022) was developed. In the internal validation test, the accuracy for predicting six key points was 0.91, 0.91, 0.92, 0.71, 0.99, and 0.99 for each point, with a single alpha of 0.707, a beta of 0.709, and a containment of 0.72. For the external validation test using images from Pusan National University Hospital, the single alpha was 0.979, the beta was 0.978, and the containment was 0.882. When using images with a quality of 7 or higher, the AI’s diagnostic accuracy was 0.974, sensitivity 0.929, and specificity 1.

### 2.3. Participants

Seventy medical students (53 men and 17 women) from Keimyung University College of Medicine participated in this study in 2022. Of these, 17 students who did not participate were excluded. Thus, 53 students finally participated in this study. All participants consented to participate and this was approved by the Institutional Review Board of Pusan University Hospital (PNU IRB no. 2301-013-123). After receiving brief instruction on DDH and the use of the software, students independently identified six key points on the ultrasound images. They then repeated the measurements on the same images with AI assistance.

### 2.4. Use of Labeling Tools

A specially customized labeling tool was used to mark the six key points. The labeling process was initiated by uploading the data to the SW, followed by the determination of the scan quality. Scan quality was assessed using a 10-point scoring system. The gross configuration was determined by 0 or 4 points, and whether the ilium was straight was determined by 0–2 points. The triradiate cartilage score was also divided from 0–2, and the presence or absence of the labrum and femoral head was scored as 0 or 1, respectively. The six key points were based on Graf’s method. The first and second key points were the parts of the bony ilium along a straight line. The third key point was at the most inferior aspect of the ilium along the line extending from the triradiate cartilage to the lower ilium, and the fourth was the outer margin of the labrum. These four key points were used to calculate alpha and beta Graf angles. The fifth and sixth key points were located at the medial and lateral aspects of the femoral head, respectively (Figure 2). Containment was calculated as b/a + b × 100 (a: from the medial of the femoral head to the center of the femoral head; b: from the center of the femoral head to the lateral of the femoral head).

### 2.5. Statistical Analysis

Statistical analysis was performed using SPSS version 21.0 (IBM Corp., Armonk, NY, USA). The measurements obtained before and after AI assistance were paired within each student. Agreement between the students’ measurements and those of an experienced pediatric orthopedic surgeon was evaluated using the intraclass correlation coefficient (ICC). The surgeon’s measurements were used as the reference standard. For comparisons of agreement scores before and after AI assistance, the Wilcoxon signed-rank test was used for paired observations. Agreement scores were categorized into four grades: Excellent (≥0.75), Good (0.60–<0.75), Fair (0.40–<0.60), and Poor (<0.40). Students with a negative agreement score for any of the evaluated parameters were excluded from the analysis. Students without paired measurements before and after AI assistance were also excluded.

Changes in the distribution of the four agreement grades before and after AI assistance were assessed using the Stuart–Maxwell test for marginal homogeneity. All statistical tests were two-sided, and a *p*-value < 0.05 was considered statistically significant.

## 3. Results

As shown in Table 1, AI assistance substantially improved agreement with the expert reference. For the alpha angle, the proportion of students classified as having excellent agreement increased from 14 (26.4%) before AI assistance to 43 (81.1%) after AI assistance, while the proportions classified as good, fair, and poor decreased accordingly. The distribution of agreement grades differed significantly before and after AI assistance (*p* < 0.001). For the beta angle, the proportion of students in the excellent agreement category increased from 36 (67.9%) to 46 (86.8%); however, the overall change in the distribution of agreement grades did not reach statistical significance (*p* = 0.073). For containment, the proportion of students classified as having excellent agreement increased from 30 (56.6%) to 45 (84.9%), with a corresponding reduction in the lower agreement categories. The distribution of agreement grades significantly improved after AI assistance (*p* < 0.001). As shown in Table 2, the mean ICC increased significantly after AI assistance for all three measurements. The mean ICC for the alpha angle increased from 0.593 ± 0.202 before AI assistance to 0.855 ± 0.156 after AI assistance (*p* < 0.001). Similarly, the mean ICC for the beta angle increased from 0.760 ± 0.169 to 0.892 ± 0.125 (*p* < 0.001), while the mean ICC for containment increased from 0.722 ± 0.201 to 0.882 ± 0.127 (*p* < 0.001). As presented in Table 3, AI assistance also improved the diagnostic accuracy based on the alpha-angle classification criterion of 60°. The accuracy increased from 0.785 with self-measurement to 0.921 with AI-assisted measurement (*p* < 0.001). In contrast, when an alpha-angle threshold of 55° was used, accuracy changed from 0.760 to 0.748, without a statistically significant difference (*p* = 0.087).

## 4. Discussion

In this study, we demonstrated that AI-assisted ultrasound measurement improved agreement with an expert reference among medical students undergoing DDH ultrasound assessment. DDH is a hip disorder that ranges from subtle acetabular dysplasia to subluxation and apparent dislocation of the femoral head, potentially resulting in hip instability [5,19]. As early diagnosis is an important factor in the management of DDH, hip ultrasonography is widely used as a primary method for screening, diagnosis, and follow-up [20]. However, ultrasound is an operator-dependent diagnostic modality that requires adequate technical experience, and interobserver variability remains a concern in the assessment of DDH [5,21]. In a previous study, the ICCs among two pediatric orthopedic professors, two orthopedic specialists, and two orthopedic residents were 0.622 and 0.054 for the alpha and beta angles, respectively (*p* < 0.001 and *p* = 0.003). Furthermore, no significant agreement was observed between professors and residents (ICC = −0.019, *p* = 0.737) or between specialists and residents (ICC = 0.066, *p* = 0.014) for the beta angle. Bar-On et al. also reported that hips classified as normal on ultrasound were classified as normal on repeated examinations in 98% of cases [20]. Although the Graf method uses specific anatomical landmarks to standardize ultrasound assessment, interobserver differences may still occur [2,20]. To address these challenges, we evaluated whether AI-assisted key-point identification could support DDH ultrasound measurement among novice users.

In this study, AI assistance substantially improved the agreement between medical students’ measurements and those of an experienced pediatric orthopedic surgeon. The proportion of students classified as having excellent agreement increased markedly after AI assistance, particularly for the alpha angle and containment. In addition, the mean ICC values increased significantly for all three measurements. These findings suggest that AI-based key-point guidance can reduce variability in ultrasound measurements among novice users and facilitate measurements that are more consistent with those of an experienced examiner. The improvement observed across the alpha angle, beta angle, and containment suggests that AI assistance may be useful not only for identifying individual anatomical landmarks but also for supporting the quantitative interpretation of Graf-based hip ultrasound examinations. Although the distribution of agreement categories for the beta angle did not reach statistical significance, the continuous ICC analysis demonstrated a significant improvement, suggesting that the overall magnitude of agreement improved even when the distribution of categorical agreement levels did not change significantly.

The improvement was particularly evident when the 60° alpha-angle threshold was used. Diagnostic accuracy increased from 0.785 with self-measurement to 0.921 with AI-assisted measurement (*p* < 0.001). In contrast, when a 55° threshold was applied, accuracy changed from 0.760 to 0.748, without a statistically significant difference (*p* = 0.087). These findings suggest that the effect of AI assistance may vary according to the alpha-angle threshold used for classification. Therefore, further studies are needed to determine how AI-assisted measurement performs across different diagnostic thresholds and clinical classification criteria.

Recent advances in artificial intelligence have demonstrated its potential to support medical diagnosis and disease screening. AI-based systems have been investigated in various clinical fields, including diabetic retinopathy, where automated image analysis can assist in disease detection and screening [9,22]. These developments support the potential utility of AI as an adjunctive tool in image-based medical assessment. In the context of DDH, AI-assisted identification of anatomical landmarks may similarly provide structured guidance for users who have limited experience with hip ultrasonography.

This study demonstrated the potential of AI-based diagnostic assistance software to improve agreement with expert measurements among medical students and may help address some of the technical difficulties associated with ultrasound-based measurement. However, several limitations should be acknowledged. First, of the 70 students initially included, 53 were included in the final analysis, which may limit the generalizability of the findings. Second, the participating students had different levels of individual ability and experience, which may have influenced their measurement performance. Third, the study evaluated medical students rather than healthcare professionals who routinely perform or interpret hip ultrasonography. In addition, the study focused on agreement with an experienced examiner rather than clinical outcomes or longitudinal diagnostic performance. Therefore, the findings should be interpreted as evidence of improved measurement agreement rather than direct evidence of improved clinical diagnostic outcomes. Future studies should evaluate the efficacy and practical utility of AI-assisted ultrasound measurement among clinicians with different levels of experience, including pediatricians, radiologists, and orthopedic surgeons. Studies conducted in real-world clinical settings and involving larger, independent cohorts will be important to determine whether the observed improvement in measurement agreement translates into improved diagnostic performance and clinical decision-making.

## 5. Conclusions

In conclusion, our findings suggest that AI-assisted ultrasound measurement may improve the accuracy of DDH assessment among non-skilled professionals. This approach may have potential as a supportive tool for DDH screening and educational training in medical institutions.

## Figures and Tables

**Figure 1:**
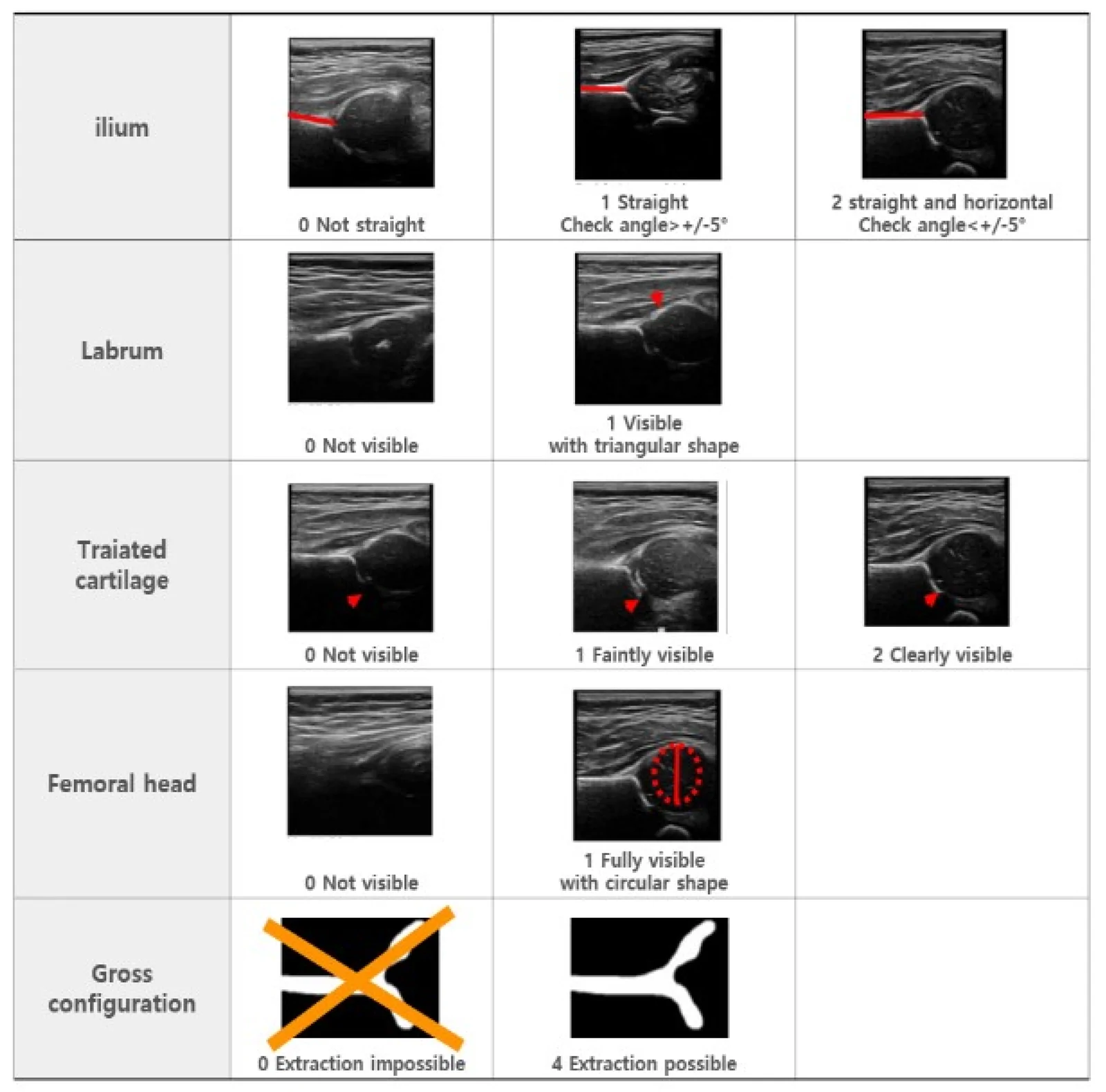
Modified 10-point scoring system. The red symbols marked on each photo are the ilium, labrum, triradiated cartilage, and femoral head, and the orange cross mark indicates cases where the gross configuration is not visible.

**Figure 2:**
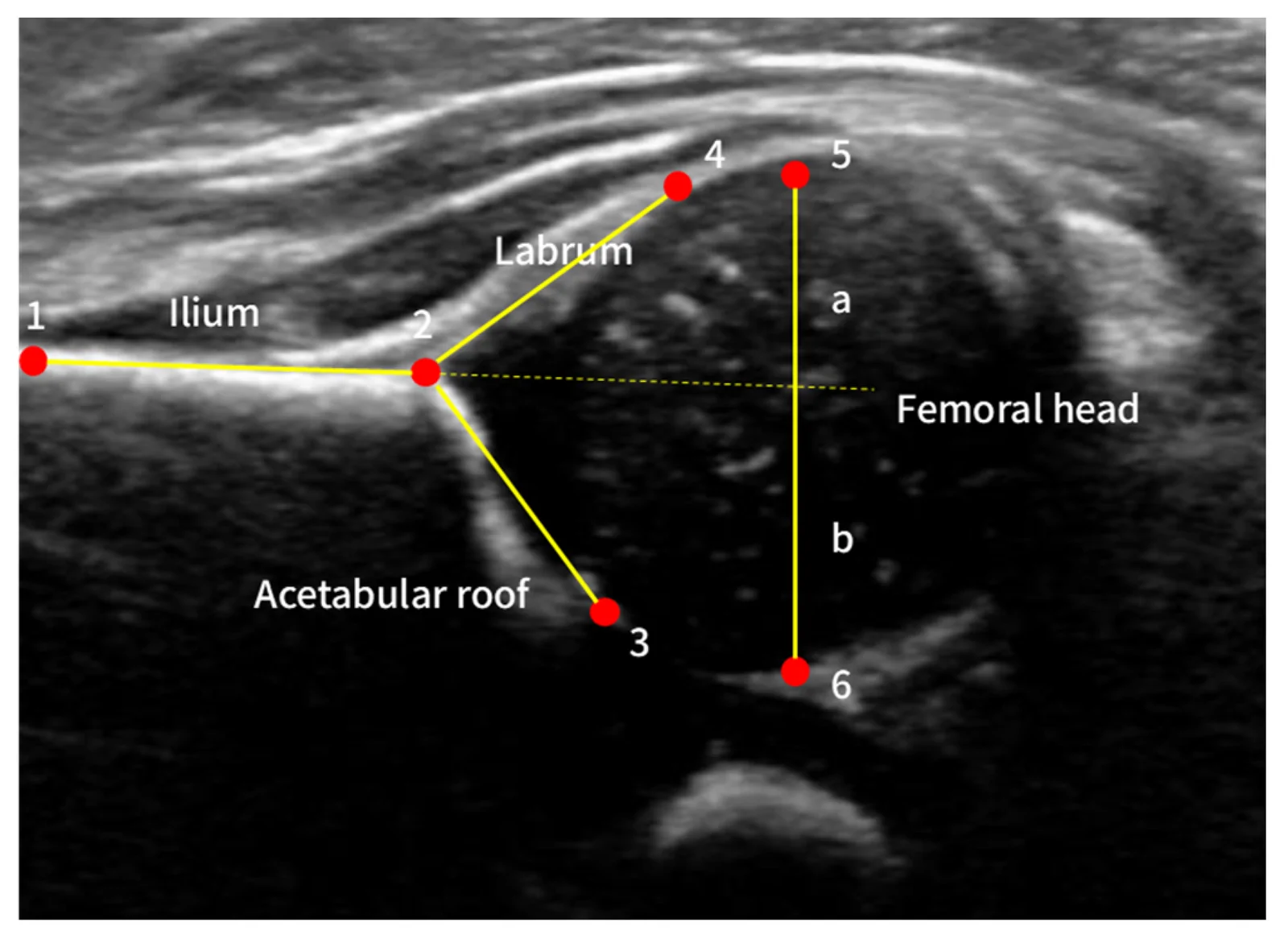
Visualization of six key points. 1; anterior superior iliac spine, 2; a nterior inferior iliac spine, 3; triradiated cartilage, 4; labrum, 5; lateral aspect of the femoral head, 6; medial aspect of the femoral head.

**Table 1:** Distribution of agreement categories before and after AI assistance.

Classification		Before AI, *n* (%)	After AI, *n* (%)	*p* Value
Alpha	Excellent	14 (26.4)	43 (81.1)	<0.001
	Good	19 (35.8)	7 (13.2)	
	Fair	9 (17.0)	2 (3.8)	
	Poor	11 (20.8)	1 (1.9)	
Beta	Excellent	36 (67.9)	46 (86.8)	0.073
	Good	10 (18.9)	5 (9.4)	
	Fair	4 (7.5)	1 (1.9)	
	Poor	3 (5.7)	1 (1.9)	
Containment	Excellent	30 (56.6)	45 (84.9)	<0.001
	Good	8 (15.1)	5 (9.4)	
	Fair	11 (20.8)	3 (5.7)	
	Poor	4 (7.5)	0 (0.0)	

*p* values were obtained using the Stuart–Maxwell test for marginal homogeneity, comparing the distribution of agreement grades before and after AI assistance. Analysis included 53 students with paired measurements.

**Table 2:** Comparison of continuous ICC scores before and after AI assistance.

Measurement	Before AI, Mean ± SD	After AI, Mean ± SD	Mean Change	Wilcoxon Signed-Rank Statistic	*p* Value
Alpha	0.593 ± 0.202	0.855 ± 0.156	0.262	20	<0.001
Beta	0.760 ± 0.169	0.892 ± 0.125	0.132	150	<0.001
Containment	0.722 ± 0.201	0.882 ± 0.127	0.16	49	<0.001

Values are presented as mean ± standard deviation. *p* values were obtained using the Wilcoxon signed-rank test for paired observations. Analysis included 53 students with complete paired measurements before and after AI assistance.

**Table 3:** Comparison of diagnostic accuracy in the excellent group using Graf’s method alpha angle criteria (55° vs. 60°).

Classification	Self-Measurement	AI SW-Assisted Measurement	*p* Value
Alpha 60	0.785	0.921	<0.001
Alpha 55	0.760	0.748	0.087

## Data Availability

The datasets generated and/or analyses during the current study are not publicly available because the data is from patients who visited the hospital but are available from the corresponding author upon reasonable request.
